# Cancer progression in COVID-19: integrating the roles of renin angiotensin aldosterone system, angiopoietin-2, heat shock protein-27 and epithelial mesenchymal transition

**DOI:** 10.3332/ecancer.2020.1099

**Published:** 2020-09-09

**Authors:** Aritra Saha, Prajna Anirvan

**Affiliations:** 1 Department of Internal Medicine, Assam Medical College and Hospital, Dibrugarh 786002, Assam, India; 2 Department of Gastroenterology, Sriram Chandra Bhanj Medical College and Hospital, Cuttack 753007, Odisha, India; a https://orcid.org/0000-0003-4705-7453; b https://orcid.org/0000-0003-4494-0865

**Keywords:** cancer progression, COVID-19, angiopoetin-2, RAAS, EMT, HSP-27 phosphorylation

## Abstract

The ongoing coronavirus disease 2019 (COVID-19) pandemic has affected millions worldwide and has been found to cause severe disease in patients with underlying comorbidities. In patients with known malignancies, in addition to constraints in routine healthcare, the risk of being susceptible to developing severe forms of the disease is of grave concern.

While follow-up studies on survivors of the severe acute respiratory syndrome (SARS) 2003 outbreak revealed increased susceptibility to infections, tumours and cardiovascular abnormalities, recent studies implicating angiopoietin 2 in induction of inflammatory intussusceptive angiogenesis and diffuse alveolar damage in COVID-19 patients raises the possibility of progression of carcinogenetic processes in patients with known malignancies. Angiotensin converting enzyme-2 (ACE-2) mediated cellular entry of SARS-Cov2 leads to receptor shedding of ACE-2 and disrupts the renin angiotensin aldosterone axis (RAAS). This augments the pro-inflammatory and proliferative effects of RAAS, while attenuating the anti-inflammatory and anti-proliferative angiotensin 1-7 /Mas pathway. Angiopoietin-2, a molecule responsible for angiogenesis and cancer progression which corelates with tumour load in certain cancers, is upregulated by angiotensin 2-AT1 Receptor axis. Tumour microenvironment—comprising of various cells, blood vessels and extra cellular matrix which express the RAAS peptides—plays a key role in cancer initiation, progression and metastasis. Angiotensin 2 induces the formation of a desmoplastic environment, favouring cancer cell growth. ACE-2 downregulation causes bradykinin accumulation which may exert its proliferative action via mitogen activated protein kinase pathways which has established roles in cancers of breast and kidney.

In addition to cytokine storm causing organ damage, acute inflammation in COVID-19 may also cause epithelial mesenchymal transition and heat shock protein 27 phosphorylation, both of which are key mediators in cancer signalling pathways.

We hypothesise that SARS-Cov2, by impacting the RAAS and immune system, has the potential to cause tumour cell proliferation, apoptosis evasion and metastasis, thereby increasing the possibility of cancer progression in patients with known malignancies.

## Introduction

With over 13 million cases and more than half a million deaths reported worldwide [1], the coronavirus disease 2019 (COVID-19) pandemic has shaken the foundations of healthcare all over the world. With the numbers continuing to rise by the day, physicians and scientists have been left scurrying to find effective therapeutic strategies to combat this pandemic. People with underlying comorbidities such as hypertension, diabetes, chronic kidney disease, chronic obstructive pulmonary disease (COPD) and malignancies seem to be more vulnerable and at increased risk of developing severe disease [2]. While the mortality rate of COVID-19 in the general population varies from less than 1% to more than 15% in different countries [3], with comorbidities, it may increase up to 25% as seen in COPD [2]. In Wuhan, with cancer as one of the comorbidities, the mortality ranged from 25% to 28.6% [4, 5], while in a subsample of 355 patients in Italy who succumbed to COVID-19, 20.3% patients had active cancer [6]. Overburdened hospitals, delayed elective surgeries and lockdown measures affecting routine cancer management, has added significantly to the problem of managing cancer patients during this pandemic [7].

Interestingly, follow-up studies on survivors of severe acute respiratory syndrome (SARS) outbreak in 2003 revealed increased susceptibility to infections, cardiovascular abnormalities and tumours of spinal cord, breast and uterus [8]. Studies have revealed angiopoietin-2 induced inflammatory intussusceptive angiogenesis along with diffuse alveolar damage in the lung specimens of COVID-19 patients [9, 10]. This raises the possibility of COVID-19 infection furthering the progression of cancer in patients with established malignancies.

In this paper, we have tried to elucidate the possible mechanisms that may lead to progression of an already existing malignancy in the setting of COVID-19.

## SARS-CoV-2, immune activation and renin angiotensin aldosterone system (RAAS)

While the pathophysiologic mechanisms of SARS-CoV2 are yet to be fully deciphered, an attempt has been made to understand the pathogenesis of COVID-19 using SARS in 2003 and middle eastern respiratory syndrome in 2012 as models [11].

SARS-CoV2 is transmitted via the respiratory route and gains access to lung epithelial cells through angiotensin converting enzyme 2 (ACE-2) receptor [12].

Viral replication and release of viral particles leads to activation of innate immune system, causing cytokine release while interaction of antigen presenting cells with major histocompatibility complex leads to the antibody formation [11]. Unregulated and uncontrolled release of cytokines may occur in COVID-19 patients eventually culminating in acute respiratory distress syndrome (ARDS) and subsequent mortality [13].

ACE-2 mediated viral entry leads to ACE-2 receptor shedding, causing an imbalance among different components of the RAAS pathways [14]. The canonical RAAS pathways, which are ACE dependent, have been described in literature [15]. In addition, the non-canonical pathway which includes molecules like AT (1–9), AT (1–7), ACE-2, AT-A, alamandine, Mas receptor (MasR), Mas-related G-protein coupled receptor type D and ACE-2 has vasodilatory, anti-proliferative, antifibrotic and anti-inflammatory effects, which counteracts the effects of the classical pathway [16].

Therefore, an increased ACE/ACE-2 ratio shifts the entire pathway from non-canonical to canonical, augmenting the deleterious effects of RAAS [14].

## Potential pathways of cancer progression in COVID-19 (Figure 1)

### Angiopoietin-2

Angiopoietin-2 level correlates well with the severity of ARDS [15, 16]. Angiopoietin-2 promotes angiogenesis and increases vascular permeability by antagonising the action of angiopoietin-1 [19]. Recent comparative studies on lungs autopsy samples of COVID-19 and 2009 H1N1 influenza patients have revealed diffuse alveolar damage in both groups [9]. However, upregulation of genes responsible for ‘intussusceptive angiogenesis’ was more predominant in the specimens of COVID-19 subjects with angiopoietin-2 being strongly implicated in these patients [9].

This assumes significance in view of the fact that angiopoietin-2 has been shown to play a key role in angiogenesis in the process of cancer progression and metastasis in patients with breast cancer [19]. Angiopoietin-2 levels also corelated with tumour load and survival in patients with cutaneous malignant melanoma [20].

Intussusceptive angiogenesis, one of the less commonly known and later discovered types of angiogenesis, is known to occur in both normal tissues and in pathological conditions including cancers like B cell non-Hodgkin lymphoma, breast cancer, renal cell carcinoma and glioblastoma and is relatively less responsive to standard anti-angiogenic therapies compared to sprouting type of angiogenesis [21].

### Heat shock protein 27 (HSP-27) phosphorylation

HSP-27 is a chaperone belonging to the family of small molecular weight heat shock proteins [22]. *In vitro* studies have revealed that inhibition of HSP-27 phosphorylation reduced the proliferation, migration and invasion of colon cancer cells, whereas xenograft studies have revealed that the same inhibition results in attenuated tumour progression [23].

HSP-27 phosphorylation in the context of COVID-19 assumes significance in view of the following observations:

#### Cytokines

*In vitro* studies have revealed that cytokines like interleukin-1 and tumour necrosis factor-alpha have the ability to phosphorylate HSP-27 [24]. There is a possibility that COVID-19 induced cytokine storm in patients with pre-existing malignancies could potentially cause HSP-27 phosphorylation and lead to tumour progression.

#### Bradykinin (BK)

Downregulation of ACE-2 increases the levels of BK [25]. BK exerts its effects through mitogen activated protein kinase (MAPK) pathway [25]. The ability of MAPK to phosphorylate HSP-27 has been documented [26]. In addition, the role of BK in mediating HSP-27 phosphorylation, albeit to a lesser extent, has also been studied [27].

### RAAS dependent pathways

#### Tumour micro-environment

The tumour micro-environment (TME) consists of extra cellular matrix (ECM), macrophages, fibroblasts, endothelial cells, inflammatory cells, etc. TME is associated with various steps in cancer progression which includes initiation, progression and metastasis [28].

A special subset of fibroblasts, known as cancer-associated fibroblasts (CAF) play a key role in cancer progression, angiogenesis, modulation of the ECM and stimulating cancer cell growth by releasing anti-inflammatory cytokines and growth factors [29].

**Figure 2.**
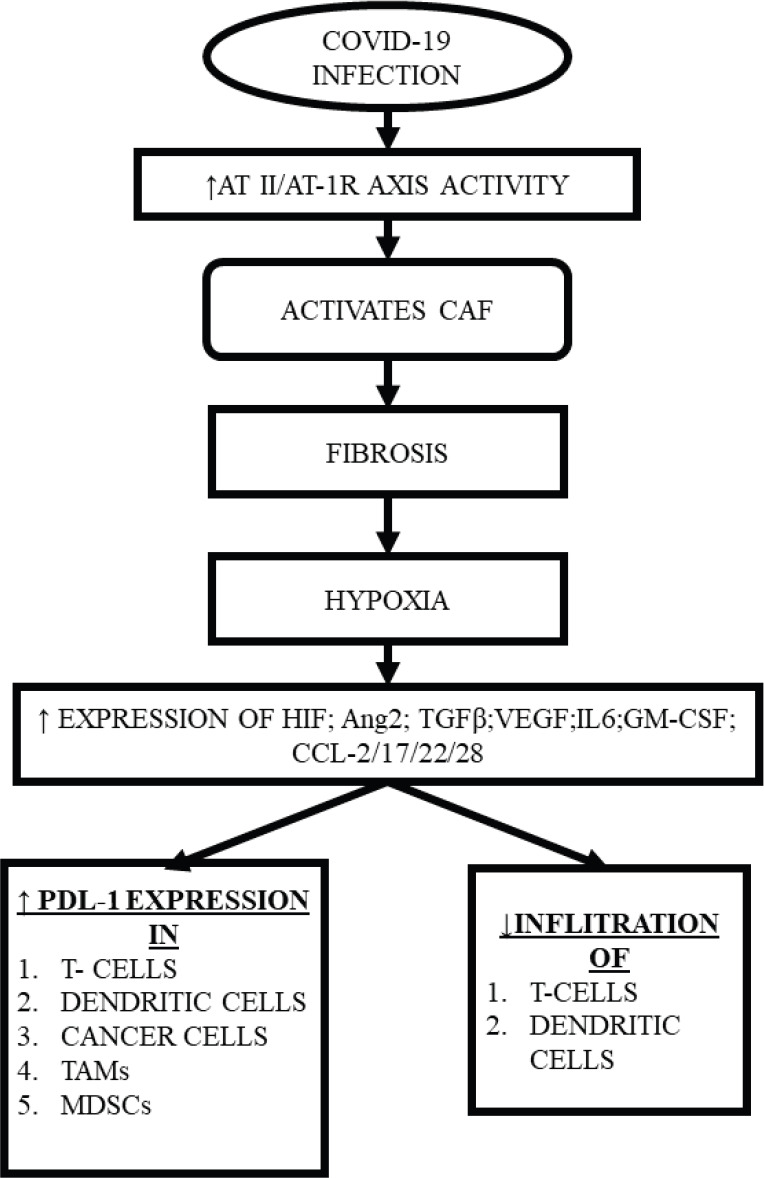
CAF induced hypoxia and subsequent consequences.

Interestingly, multiple components of TME, including CAF, also express RAAS components, with RAAS modulating the actions of CAF through multiple mechanisms creating a desmoplastic environment, which in turn, reduces the penetrance of T-cells, leading to accumulation of aberrant cells [30], while CAFs individually can inhibit the T-cell and natural killer (NK) cell function and promote the formation of a pro-inflammatory tumorigenic niche [31]. Upon activation, CAF also increases the synthesis of collagen-1, promoting fibrosis that induces hypoxia by compressing the blood supply [32], while hypoxia itself may lead to expression of immune-inhibitory molecules like programmed death ligand 1, reduction in the potential of the tumour killing cells and reprogramming of the macrophages into immune-suppressive type [28, 30] (Figure 2).

#### Angiotensin-2 angiotensin-type1 receptor axis

As already mentioned, SARS-CoV2 entry into the cell downregulates the ACE-2 enzyme, increasing the activity of AT II, by shifting the RAAS towards the Angiotensin-Type 2 (AT II)-AT-1R axis.

*In vitro* studies on human small lung cancer cells have revealed that AT II is responsible for the formation of cancer stem cells, which has been implicated in cancer initiation, metastasis and relapse [34].

Apart from that, other *in vivo* and *in vitro* studies have revealed that AT II- AT-1 receptor axis promotes angiogenesis in solid tumours such as in the cancers of ovary, breast and bladder via vascular endothelial growth factor (VEGF) upregulation [35, 36]. Studies have also demonstrated that concentration of AT-1R correlated with the expression of VEGF, VEGF receptor and micro-vessel density in a tumour [37]. VEGF, apart from angiogenesis, also increases vascular permeability, which further aggravates hypoxia and potentiates detrimental effects in a TME [30, 33]. Another study used AT-1R-positive MDA-MB-231 human breast cancer cells to show that AT II increased the cell migration and expression of matrix metalloproteinase, via phosphatidyl inositol 3 kinase, nuclear factor-kappa beta (NF-KB) and Akt pathways, while the cellular migration reduced on using the specific inhibitors of each pathway thus suggesting blocking these pathways could be used for treating metastatic breast cancer [38].

#### Bradykinin

The high molecular weight kininogen is metabolised by the kallikrein into BK and des arginine 9-bradykinin (DABK) —the active metabolite of bradykinin. While the former product binds to Bradykinin B2 receptor (BKB2R), the later binds to BKB1R [39]. ACE-2 hydrolyses DABK [41].

Downregulation of ACE-2 leads to accumulation of DABK, which binds to BKB1R to exert its pro-inflammatory actions, which may partly be responsible for the features of ARDS in COVID-19 patients [42].

Apart from inflammation, BK also has a role in tumour progression, as evident by the expression of BKB1R in cancers of breast, kidney, stomach, oesophagus, malignant mesothelioma, cervix, prostate and expression of BKB2R in the HNSCC (head and neck squamous cell carcinoma), osteosarcomas, cancers of endometrium, kidney, stomach and pituitary gland [25]. BK mediates its proliferative action via MAPK and extracellular signal related kinases 1 and 2 [43].

#### Suppression of ACE-2/Ang-(1–7)/MasR axis

Downregulation of ACE-2 secondary to COVID-19 infection leads to suppression of the non-canonical pathway- ACE-2/AT-(1–7)/MasR axis [14]. *In vitro* and *in vivo* murine studies have revealed that AT (1–7) via MasR exerts anti-proliferative effect in angiogenesis, tumour-associated inflammation, fibrosis, and metastasis [44].

Studies have revealed that cancers of breast, liver, lungs with increased ACE-2 expression had lower cellular proliferation rates [45], but this data may vary with the type of cancer.

In contrast, some studies have shown that patients with hepatocellular carcinoma with high levels of ACE-2 had a longer survival time [44], whereas other studies have suggested that decreased ACE-2 may be suggestive of cancer in a diabetic patient [46].

**Figure 3.**
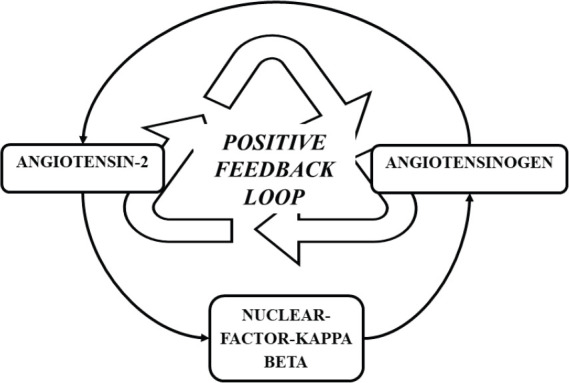
Positive feedback loop formed by NF- κB, sustaining the formation of AT II.

RAAS activation secondary to COVID-19 infection is a temporary event and the duration of infection may not be significant enough to induce carcinogenesis. However, murine studies have revealed that AT II exerts its inflammatory action via NF-KB and the same study revealed that NF-KB is a mediator for the re-synthesis of angiotensinogen, the precursor of AT II [47]. Thereby, a positive feedback loop is formed (Figure 3), augmenting the inflammatory action of AT II and possibly potentiating the inflammatory processes in individuals with preexisting malignancies.

### Epithelial mesenchymal transition (EMT)

EMT is the process by which polarised epithelial cells upon receiving certain signals, undergo biochemical changes to acquire the properties of mesenchymal cells [48]. At the primary tumour site, acquiring mesenchymal properties allows the cancer cell to get rid of the epithelial junctions and degrade the extra cellular matrix which aids the same cell to disseminate [49]. The pivotal role of EMT in metastasis is further corroborated as the circulating tumour cells (CTC) express the markers for both epithelial and mesenchymal cells, while in refractory or progressive cases the expression of the mesenchymal markers is higher [50]. While mesenchymal state of the disseminated cancer cells is capable enough to exhibit the last step of colonisation by forming macro-metastases, certain cancer types require the epithelial properties to form macro-metastases and therefore undergo mesenchymal to epithelial transition to accomplish the same result [51].

Studies have revealed that inflammatory tumour micro-environment could be responsible for progression of colorectal cancer [52]. Other studies have corroborated this by showing that an exposure to inflammatory micro-environment could in fact cause EMT [53]. Entry of SARS-CoV2 augments the Ang II- AT1R axis promoting the formation of a pro-inflammatory tumour niche, secondary to reduced penetrance by T cell and NK cells (refer to Section Tumour micro-environment). While it is primarily chronic inflammation that is believed to be responsible for inducing neoplastic changes, a murine study has showed that acute inflammation can lead to progression of neoplasia by via EMT and CTC, as evident by the increased expression of their respective markers [54].

Apart from EMT being induced by inflammation, infection with oncogenic viruses like Epstein Barr virus, human papilloma virus, hepatitis C virus have established role in triggering cancer initiation and progression via EMT along with other pathways [55, 56].

### Lysophosphatidyl inositol (LPI)

Even though no data for the elevated levels of LPI in patients of COVID-19 is available in literature as of now, a follow up study revealed that survivors of SARS 2003 had deranged lipid and glucose metabolism, with elevated levels of LPI, probably secondary to elevated levels of PI (phosphatidyl inositol) [8]. Multiple studies have revealed that lysophosphatidyl inositol-G protein coupled receptor 55 axis has a role in cell proliferation, migration and tumourigenicity [57].

However, it must be mentioned that the same follow up study disclosed that elevated levels of LPI could be due to treatment with steroids, which has not been advocated in treating COVID-19 due to its controversial effects [58].

## Conclusion

The extensive involvement of various systems secondary to inflammation and possible involvement of RAAS is evident in COVID-19. This leads to a cascade of events at the molecular level and it is quite possible that COVID-19 positive cancer patients may be at an increased risk of cancer progress and metastasis, with the risk being more in those cancer patients with underlying chronic inflammatory diseases.

However, this requires rigorous follow-up studies on such patients with meticulous monitoring of disease progression and special emphasis on the inflammatory changes and role of RAAS components.

## List of abbreviations

- COVID-19: coronavirus disease 2019
- SARS-CoV2: severe acute respiratory syndrome coronavirus 2
- RAAS: renin angiotensin aldosterone system
- AT: angiotensin
- AT II: angiotensin 2
- AT-R: angiotensin receptor
- ACE: angiotensin converting enzyme
- MasR: Mas receptor
- TME: tumour micro-environment
- ECM: extracellular matrix
- NK cell: natural killer cells
- VEGF: vascular endothelial growth factor
- EMT: epithelial to mesenchymal transition
- HSP: heat shock protein
- BK: bradykinin
- BK-B1/2R: bradykinin B1/B2 receptor
- DABK: des-arginine9 bradykinin
- CAF: cancer-associated fibroblasts
- CTC: circulating tumour cells
- PI: phosphatidyl inositol
- IL: interleukin
- MAPK: mitogen activated protein kinase

## Conflicts of interest

The authors have none to declare.

## Authors’ contributions

AS: conceptualisation, data curation, writing—original draft preparation.

PA: critical revision, data curation, writing—review and editing.

The final version of the article, including the authorship list, was approved by all of the authors.

## Funding declaration

No funding was received.

## Figures and Tables

**Figure 1.**
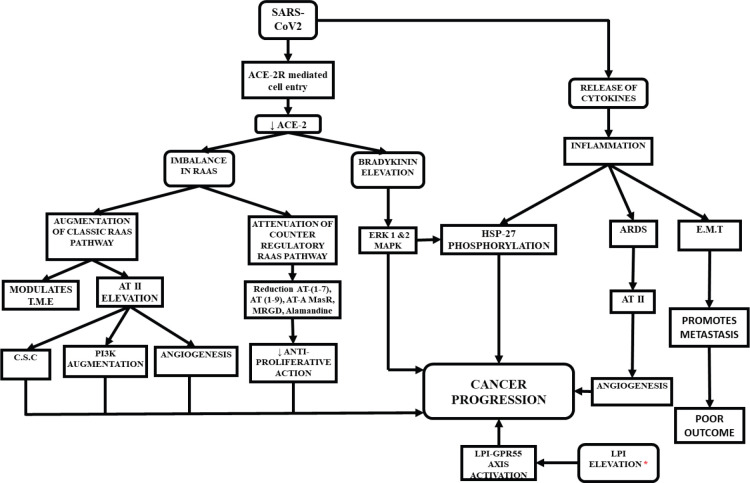
Possible ways of cancer progression in COVID-19 patients. Elevated levels of LPI was noticed in recovered patients of SARS-2003.
